# Redundancy, resilience, and host specificity of the ruminal microbiota: implications for engineering improved ruminal fermentations

**DOI:** 10.3389/fmicb.2015.00296

**Published:** 2015-04-10

**Authors:** Paul J. Weimer

**Affiliations:** ^1^US Dairy Forage Research Center, US Department of Agriculture – Agricultural Research ServiceMadison, WI, USA; ^2^Department of Bacteriology, University of WisconsinMadison, WI, USA

**Keywords:** fermentation, host specificity, redundancy, resilience, rumen

## Abstract

The ruminal microbial community is remarkably diverse, containing 100s of different bacterial and archaeal species, plus many species of fungi and protozoa. Molecular studies have identified a “core microbiome” dominated by phyla Firmicutes and Bacteroidetes, but also containing many other taxa. The rumen provides an ideal laboratory for studies on microbial ecology and the demonstration of ecological principles. In particular, the microbial community demonstrates both redundancy (overlap of function among multiple species) and resilience (resistance to, and capacity to recover from, perturbation). These twin properties provide remarkable stability that maintains digestive function for the host across a range of feeding and management conditions, but they also provide a challenge to engineering the rumen for improved function (e.g., improved fiber utilization or decreased methane production). Direct ruminal dosing or feeding of probiotic strains often fails to establish the added strains, due to intensive competition and amensalism from the indigenous residents that are well-adapted to the historical conditions within each rumen. Known exceptions include introduced strains that can fill otherwise unoccupied niches, as in the case of specialist bacteria that degrade phytotoxins such as mimosine or fluoroacetate. An additional complicating factor in manipulating the ruminal fermentation is the individuality or host specificity of the microbiota, in which individual animals contain a particular community whose species composition is capable of reconstituting itself, even following a near-total exchange of ruminal contents from another herd mate maintained on the same diet. Elucidation of the interactions between the microbial community and the individual host that establish and maintain this specificity may provide insights into why individual hosts vary in production metrics (e.g., feed efficiency or milk fat synthesis), and how to improve herd performance.

## Introduction

The rumen is the characteristic defining feature and most voluminous digestive organ of ruminant animals, and the microbial fermentation that occurs within is largely responsible for providing the energy and protein needs of the animal, in the form of volatile fatty acids (VFA) and microbial cell protein, respectively (Hungate, 1966). Because the ruminal fermentation is of central importance in ruminant nutrition, the prospects of manipulating the fermentation to improve the performance of the predominant ruminant livestock species (cattle, sheep and goats) has long attracted the attention of both microbiologists and animal scientists (Chalupa, 1977). In addition to its practical importance, the rumen is an ideal laboratory for elucidating fundamental principles of microbial ecology, for several reasons: i) The organ itself is of manageable size, and is contained within a unique animal unit; ii) access can readily provided by cannulation surgery; iii) the experimentalist can dictate the types and amounts of inputs (feed and water), and can accurately measure outputs; and iv) assembling a number of habitats for replicated studies is as simple as gathering the desired number of animal units. None of these useful properties are as easily embodied in other commonly studied microbial habitats (e.g., soils, lakes, etc.). And while it is clear that the ruminal microbial community is rich in terms of both its biomass density and its species diversity, this community operates under the same ecological principles as do the microbial communities in other habitats (Weimer, 1998).

Central to attaining the goal of improving the ruminal fermentation are fundamental questions regarding variability in the composition and activities of the ruminal microbiota, and the extent to which these factors impact overall animal performance. Historically attempts at ruminal manipulation have focused on using chemical agents (reviewed by Chalupa, 1977) or enzymes (reviewed by Beauchemin et al., 2003) as feed additives. However, recent advances in our understanding of the microbial community have allowed us to formulate strategies based on microbial agents (e.g., probiotics) that might contribute to a re-engineering of community dynamics and activities. These microbially based approaches, particularly those based on Domain Bacteria, are the subject of this analysis.

## The Ruminal Microbiota

The ruminal microbial community consists of two groups of procaryotes (bacteria and archaea) and two groups of eucaryotes (protists and fungi). Bacteria and protists together account for well over 90% of the microbial biomass, and the bacteria in particular have been the focus of most quantitative studies on community composition.

Following the development of culture techniques suitable for strictly anaerobic bacteria, extensive efforts were made by microbiologists—particularly Hungate and others – toward isolation and characterization of ruminal bacteria. The vast majority of isolates were classifiable into about two dozen species, but even then it was noted (by comparison of colony counts in agar roll tubes versus direct enumeration of cells under the microscope) that only ∼8% of the bacterial community was cultivable using standard anaerobic techniques and media (Bryant and Burkey, 1953), although later refinements in media and technique improved this value somewhat (Bryant and Robinson, 1961; Leedle and Hespell, 1980). The development of molecular methods for characterizing microbial communities by sequencing genes for small-subunit rRNA genes, and more recently by metagenomic analysis employing next-generation sequencing (NGS), have provided a fuller appreciation for the very large number of bacterial species in a typical rumen. Over the past 5 years there have been over 30 publications that have quantified the microbial community composition at different taxonomic levels based on NGS technology alone; these studies have been well summarized in the recent review of McCann et al. (2014). It is clear from these studies that community composition data are influenced by a variety of factors which can be broadly grouped into those resulting from authentic differences in composition versus those in which the community composition results were influenced by the experimental methods employed (**Table 1**). The effect of methodology was highlighted by the landmark study of Henderson et al. (2013), who showed dramatic differences in the yield, quality, and taxonomic distribution of DNA that resulted from the use of different DNA isolation methods. Techniques that employed bead-beating of samples, particularly with phenol present during the beating step, gave superior yields of high-quality DNA compared to methods that employed commercial kits routinely used for DNA isolation from soils or feces. Of particular importance was their demonstration that, upon analysis of sequences by Titanium 454 pyrosequencing, isolation methods that gave low DNA yields also gave community compositions quite different from those obtained using high-yield isolation methods.

**Table 1:** Factors that can affect the outcome of microbial community analysis in the rumen, with references for studies in which individual factors were examined systematically.

Factor	Reference
*Factors resulting from authentic differences in the subject*	
Animal type (species, breed, age, stage of ruminal development)	Lee et al. (2012), Li et al. (2012), Wu et al. (2012), Henderson et al. (2013), Rey et al. (2014)
Diet (ration ingredients and chemical composition)	Numerous studies^a^
Phase of ruminal contents (liquid, suspended solid, ruminal mat, epimural)	Numerous studies^b^
Plane of nutrition	Mohammed et al. (2012b)
Time of sample collection relative to time of feed presentation	Li et al. (2009), Welkie et al. (2010)
Ruminal pH	Khafipour et al. (2009), Palmonari et al. (2010), Mohammed et al. (2012b), Petri et al. (2013a,b)
Host specificity (animal individuality)	Weimer et al. (2010a,b), Jami and Mizrahi (2012), Mohammed et al. (2012b), Zhou et al. (2012)
*Factors resulting from study methodology*	
Method of sample collection (ruminal cannula, stomach tube)	Lodge-Ivey et al. (2009), Henderson et al. (2013)
Site of sampling (cranial/caudal, dorsal/ventral)	Li et al. (2009)
DNA isolation method	Henderson et al. (2013)
DNA analysis method (ARISA, qPCR, tRFLP, clone library construction, NGS)	de Menezes et al. (2011), Mohammed et al. (2014)

*^a^Tajima et al. (2001), Callaway et al. (2010), Fernando et al. (2010), Pitta et al. (2010), Weimer et al. (2010b), Welkie et al. (2010), de Menezes et al. (2011), Khafipour et al. (2011), Broadway et al. (2012), Mohammed et al. (2012a), Ramirez et al. (2012), Wang et al. (2012), Petri et al. (2013a,b), Zened et al. (2013), Zhang et al. (2013), Mohammed et al. (2014).*

*^b^Sadet et al. (2007), Pitta et al. (2010), Welkie et al. (2010), de Menezes et al. (2011), Fouts et al. (2012), Lee et al. (2012), Mohammed et al. (2012a, 2014), Henderson et al. (2013), Petri et al. (2013a).*

Further complications result from the use of different methods of analyzing the isolated DNA. Quantitative real-time PCR studies with genus-specific primers suggest that the genus *Prevotella* can constitute around half of the total bacterial 16S rRNA gene copy number (Stevenson and Weimer, 2007), a proxy for relative population size. By contrast, most studies that have employed pyrotag sequencing suggests that the phylum Bacteroidetes (which includes *Prevotella* and many other genera), while of major abundance, are substantially outnumbered by members of the phylum Firmicutes (de Menezes et al., 2011; Henderson et al., 2013; Mohammed et al., 2014). Similar disparities are observable by cross-comparison of different studies at almost any phylogenetic level. Overall, then, we must be cautious in interpreting quantitative abundance data across studies, and instead should focus primarily on differences among animals, treatments, and time-dependent dynamics within individual studies, or perhaps across studies that at least used similar methods of DNA isolation and analysis (Henderson et al., 2013).

One of the major problems in associating specific animal production responses with microbial community composition is that a substantial number of animals are typically required to demonstrate a statistically significant effect of treatment (e.g., diet), but the number of animals available for microbiological studies (e.g., ruminally cannulated cows for optimal sampling at a similar physiological state) is often limited. One approach to this dilemma is to conduct production experiments with a large number of cows, a small subset of which meet the desired sampling criteria (de Menezes et al., 2011), and then verify a lack of differences in production metrics between the subset of animals and the larger set. Such experimental designs may not be appropriate for all studies, however. For example, ruminally cannulated cows that provide optimal sampling logistics may not be practical for methane emissions measurements due to potential losses of fermentation gases from the vicinity of the cannula (de Menezes et al., 2011; Beauchemin et al., 2012), though this would not be an issue for animals confined to chambers for collection of gaseous emissions.

Despite the caveats listed above with regard to methodologies, it is clear that molecular approaches, particularly NGS, have tremendously expanded our appreciation for the richness and complexity of the ruminal microbial community. As these quantitative phylogenetic assessments mount, we must tackle the challenge of relating this information to the physiologies and interrelationships of the different species. This will demand that we distinguish between microbial populations and their level of metabolic activity, a problem elegantly addressed using such novel methodologies as simultaneous DNA/cDNA quantification (Lettat and Benchaar, 2013) and metatranscriptomic profiling (Dai et al., 2015). Additionally, it will ultimately be necessary to isolate and characterize new microbial species, particularly phylotypes revealed to be abundant but which have heretofore escaped cultivation in the laboratory (Kobayashi, 2006).

## Ecological Properties of the Ruminal Microbiome

### Redundancy

With regard to the ruminal microbial community, we can define *redundancy* as the overlapping distribution of physiological capabilities across multiple microbial taxa. Conceptually, redundancy with respect to catabolism can be inferred from a comparison of the number of degradable substrates (or, for polymeric substrates, the number of different monomeric units and different linkages between monomeric units) in the feed on the one hand, and the number of species available to carry out the degradations. Despite the complexity of feeds ingested by the ruminant, there are a relatively modest number of potential “degradation points” (see **Table 2 and 3** for a partial listing of biopolymers and soluble substrates, respectively). By contrast, the number of microbial species in the rumen is enormous. For example, in an oft-cited study Kim et al. (2011) used rarefaction analysis of 16S rRNA sequences archived in the Ribosomal Database Project to predict that 99.9% species coverage in the rumen would be obtained from 78,218 bacterial and 24,480 archaeal sequences, and most NGS studies have obtained 100s–1000s of operational taxonomic units (OTUs, a proxy for species) per individual ruminal sample. Dividing the large number of species by the relatively small number of degradation points provides a clear indication that, on average, there are many different species that can potentially contribute to the degradation of each substrate or linkage. Moreover, because many species can participate in the degradation of multiple substrates or attack multiple linkages within biopolymers, it is clear that the ruminal community is, from a metabolic standpoint, highly redundant in its composition. Although there are no specific surveys that have determined the number of species capable of degrading individual substrates in the rumen, it appears that catabolic redundancy is skewed heavily toward the most abundant and degradable substrates (or, for biopolymers, their monomeric units), and it would be surprising indeed if the number of species capable of degrading glucose did not greatly outnumber those capable of degrading, for example, oxalate. It thus appears that the ruminal community is composed of a mix of generalists that compete for a large number of abundant substrates, and specialists that face much less competition for a relatively smaller number of typically less abundant substrates. The redundancy of the ruminal microbial community is further suggested by the fact that, within studies, considerable changes in community composition often do not translate into changes in fundamental fermentation metrics such as pH, VFA concentrations or molar proportions of VFA (Welkie et al., 2010; Sandri et al., 2014). It must be borne in mind; however, that lack of significant differences in fermentation variables may reflect, at least in part, the use of a small number of animals per experimental treatment.

**Table 2:** Polysaccharides degraded by mixed ruminal microbes and pure culture of ruminal bacteria.

*Structural polysaccharides*	Structure	Mixed culture	Pure culture
Cellulose	β-1,4-glucan	Waldo et al. (1972), Van Soest (1973)	Hungate (1966)
Homoxylan	β-1,4-xylan	Weimer et al. (2000)	Suen et al. (2011)
Arabinoxylan	β-1,4-xylan with α-1 → 3 arabinose substituents		Hespell and Cotta (1995)
4-O-Methylglucuronoxylan	β-1,4-xylan with methylglucuronic acid substituents		Dehority (1965)
Pectins (forage)		Gradel and Dehority (1972)	Gradel and Dehority (1972)
Pectins (citrus)	Methoxylated α-1,4-galacturonic acid		Gradel and Dehority (1972)
Xyloglucan	β-1,4-glucan with β-1,6 xylose substituents		Suen et al. (2011)
*Storage polysaccharides*			
Starch	α-1.4-glucan	Mertens and Lofton (1980)	Cotta (1988)
	α-1,4 glucan with a-1,6 branching at ∼3–4% of residues		
Fructans	2,1-fructan with 1 → 2 glucosyl substituents		Ziolecki et al. (1992)
Glucomannan	β-(1 → 4)-linked mannose and glucose in a ratio of 1.6:1		Suen et al. (2011), Christopherson et al. (2014)
Laminarin	β(1 → 3):β(1 → 6) glucan (ratio of 3:1)		Teather and Wood (1982)
Lichenan	Repeating β-1,3 and β-1,4 glucan		Christopherson et al. (2014)

*Where possible, references were selected to provide a spectrum of compounds within each class, or a spectrum of taxonomy of the degradative strains.*

**Table 3:** Selected examples of reports of soluble substrate degradation by mixed ruminal microbes and pure culture of ruminal bacteria.

Nutritional strategy	Substrate class	Substrates^a^	Reference (mixed culture)	Reference (pure culture)
Generalist Specialist	Aldohexoses	Glucose, galactose, mannose	Hungate (1966)	Hungate (1966)
	Aldopentoses	Xylose, arabinose, ribose	Heald (1952)	Rasmussen (1993)
	Protein components	Amino acids, di-, and tri-peptides	Russell (2002)	Eschenlauer et al. (2002)
	Disaccharides	Cellobiose, lactose, maltose,	Heald (1952)	
	Carbohydrate oligomers	Cellodextrins,		Russell (1985), Shi and Weimer (1996)
		Maltodextrins	Kim et al. (1999)	
		Xylodextrins		Cotta (1993)
	Nucleic acids	DNA, RNA, nucleotide bases	Jurtshuk et al. (1958)	Cotta (1990)
	Ketohexoses	Fructose		
	Deoxyhexoses	Rhamnose, fucose, 2-deoxyglucose		Rasmussen (1993)
	Primary alcohols	Methanol, ethanol	Pol and Demeyer (1988), Pradhan and Hemken (1970)	Genthner et al. (1981)
	Sugar alcohols, polyols	Glycerol, mannitol, 1,2-propanediol	Lee et al. (2011)	Czerkawski et al. (1984)
	Cyclitols	*myo*-inositol, pinitol, quebrachitol	Lowry and Kennedy (1995)	
	Dicarboxyllic acids	Malonate, succinate, malate, fumarate	Russell and Van Soest (1984)	
	Hydroxyacids	Lactate, malate		Hino et al. (1994)
	Monolignols		Besle et al. (1995)	
	Phenolic acids	Ferulic acid, p-coumaric acid		Chesson et al. (1982), Long et al. (2003)
	Tricarboxylic acids	Citrate, aconitate, tricarballylate	Russell and Van Soest (1984)	
	Uronic acids	Galacturonic acid, glucuronic acid	Heald (1952)	
	Tannins			Nelson et al. (1998)
	Urea	Urea		Cook (1976)
	Amines	Cadaverine, histamine, putrescine, tyramine	Van Os et al. (1995)	
	Inorganic electron acceptors	Nitrate, sulfate, arsenate	Herbel et al. (2002), Van Zijderveld et al. (2010)	
	Hydroxyaromatic compounds	Phloroglucinol		Tsai and Jones (1975), Patel et al. (1981)
	Flavonoids	Rutin, quercitin, naringin, hesperidin	Simpson et al. (1969)	
	Plant toxins	Allyl cyanide	Duncan and Milne (1992)	
		Fluoroacetate	Camboim et al. (2012)	
		Mimosine	Jones and Megarrity (1986)	Allison et al. (1992)
		Nitro-1-propanol, nitropropionate		Majak (1992), Anderson et al. (1996)
		Oxalate	Belenguer et al. (2013)	Allison et al. (1985)
	Mycotoxins	Aflatoxin, ochratoxin, zearalenone	Kiessling et al. (1984)	
	Xenobiotic compounds	TNT, RDX	Fleischmann et al. (2004), Li et al. (2014)	

*Substrates are arranged in rough order of their abundance in feeds and forages, which roughly parallels their degradability across a spectrum of nutritional strategies from generalists to specialists. Where possible, references were selected to provide a spectrum of compounds within each class, or a spectrum of taxonomy of the degradative strains. For pure cultures, not all individual compounds listed were degraded by all of the pure cultures examined.^a^Phloroglucinol, 1,3,5-trihydroxybenzene; RDX, Hexahydro-1,3,5-trinitro-1,3,5-triazine; TNT, 2, 4, 6-trinitrotoluene.*

Aside from these generalizations, a more detailed understanding of redundancy is not easily won, for we are quickly mired in the difficulty of assigning *in situ* function to a very large number of species, only a few of which have been cultured in the lab. For example, two of the most abundant bacterial genera in the rumen are *Prevotella* and *Butyrivibrio*, and each rumen typically contains dozens to hundreds of OTUs from each of these genera, whose rRNA sequences vary sufficiently that current taxonomic fashion would classify into a large number of separate species (Pitta et al., 2010; Li et al., 2012; Mohammed et al., 2014). Cultured representatives of these genera display extremely broad degradative capabilities: hydrolysis of proteins and peptides; hydrolysis of starch and many hemicelluloses, and fermentation of many amino acids and most sugars (Kelly et al., 2010; Willems and Collins, 2011). Many of these capabilities can reside within a single bacterial strain. So while it is clear that representatives of these genera can participate in the conversion of a broad array of substrates, what is not clear is which particular degradative capability any particular species or strain might be carrying out *in situ*, in the presence of a large number of potential competitors and symbionts. The H_2_-oxidizing, CO_2_-reducing acetogens provide an object lesson in this regard: Such species have been isolated from the rumen (although they are not abundant there) but labeling studies have shown that essentially no acetate is produced from CO_2_ in the rumen (LeVan et al., 1998). Moreover, theoretical calculations have shown that these acetogens would have difficulty competing with methanogens for available H_2_, as suggested by their slower growth rates, poorer affinity for H_2_, and smaller free energy yield per mol of H_2_ consumed (Ungerfeld, 2013). Because CO_2_-reducing acetogens are very versatile catabolically, it has been suggested that their presence in the rumen reflects their ability to subsist by degrading a wide range of organic substrates, rather than by reduction of CO_2_ with H_2_ to acetate (Rieu-Lesme et al., 1996). How this contention might be proven remains at this point rather elusive.

### Resilience

Like any ecosystem, the organisms of the rumen respond to perturbation by internal or external forces. These forces may be physical (e.g., a change in temperature), chemical (e.g., ruminal pH, a change in diet composition, or an introduction of a plant toxin in the feed), or biological (e.g., the input of a non-native microbe). Perturbations may differ in intensity, frequency and duration, and these differences play a major role in determining the nature of the microbial response. Some perturbations are such common features of an animal’s existence that they are taken for granted (e.g., variations in meal patterning or water consumption). Others are less obvious, such as gradual change in forage quality in a grazing plot over time.

The ruminal response to these internal and external perturbations can be examined using concepts developed from the field of macroecology. Over the years a complex and often contradictory terminology has evolved among ecologists to describe these responses. For our purposes, we will use the terminology of Westman (1978; see **Table 3**). Responses to perturbation can be described in terms of the system’s *inertia* and its *resilience.* Inertia refers to system stability (i.e., how well it resists change), while resilience is a reflection of how the system responds once it has been changed. As discussed by Westman (1978), resilience has four components that describe the extent to which, and the path by which, an original state may be restored (**Table 4**). In macroecology, the properties of inertia and resilience are typically (and most easily) examined at the level of an individual species, and are famously illustrated by particular examples of population levels of these species over time or across spatial domains (for example, the spruce budworm in Canadian boreal forests, or whitefish in the Great Lakes; see Holling, 1973). In habitats of high species diversity, interactions among organisms become more and more complex, and unraveling the factors that underlie inertia and resilience becomes progressively more difficult for individual species and more difficult yet for entire communities. Nevertheless, it is useful to extend these concepts from classical ecology to the microbial world, including the highly diverse microbial community of the rumen, as this provides a theoretical underpinning of discussions on how successfully, and under what conditions, the ruminal fermentation might be manipulated.

**Table 4:** Characteristics that describe the stability and adaptability of the ruminal microbial community.

Characteristic	Definition^a^	Likely status in the rumen
nertia	Resistance to change	High, based on dosing studies
Resilience	Ability to restore its structure following acute or chronic disturbance	High, based on exchange studies
*Components of resilience:*			
Elasticity	Rapidity of restoration of a stable state following disturbance	Relatively high, based on exchange studies
Amplitude	Zone from which the system will return to a stable state	Very high, based on exchange studies
Hysteresis	Degree to which path of restoration is an exact reversal of path of degradation	Unknown
Malleability	Degree to which stable state established after disturbance differs from the original steady state	Low

*^a^Verbatim definitions of Westman (1978).*

Redundancy and resilience are two concepts from macroecology that appear to apply in microbial ecology as well. Other concepts may not be quite as readily transferred, at least for the ruminal habitat. For example, while the rumen may have a “core microbiome,” it is not clear whether any of its members represent *keystone species,* i.e., species that have a disproportionately large influence on the ecosystem relative to their abundance, and whose disappearance would imperil ecosystem function (Mills et al., 1993). Clearly there are keystone functional groups of microbes (e.g., fibrolytics and methanogens), but within the rumen there are multiple species representing each of these groups, and this redundancy makes it unlikely that any one species would have a role in the habitat that would be sufficiently essential and irreplaceable to merit the designation of keystone species.

### Host Individuality

Producers, especially those holding small herds who spend a lot of time interacting with their animals, are well aware of behavioral and production differences among individuals. An intriguing question is whether or not these inter-animal differences are a reflection of, or are even caused by, differences in the composition of the ruminal microbial community. The potential for a host-specific microbiological uniqueness of the ruminant was first noted in the protozoal community (Kofoid and MacLennan, 1933; Eadie, 1962), and much later in the fibrolytic bacterial community (Weimer et al., 1999), prior to the development of advanced molecular tools to characterize the gut community. The concept of host microbiome individuality has now achieved substantial attention, primarily as a result of recent studies of the human gut microbiome. Such studies have revealed that the human gut contains a “core microbiome” (i.e., a set of taxa present in all animals in the study), but also a large number of taxa whose presence or abundance varies among hosts. In numerous cases, the human gut microbiome has been shown to vary in a consistent manner with such clinical conditions as obesity or various intestinal maladies that may be grouped under the collective term *dysbiosis*. These sorts of studies have proliferated into a kind of microbiological cottage industry, and despite the welter of breathless press releases for public consumption, we are only occasionally reminded (e.g., by Hanage, 2014) that the “conclusion” of these studies have almost always been based on association, rather than on a rigorous demonstration of cause and effect.

As in the case of the human GI tract, it appears that the rumen of cattle (and probably other ruminant species) contains a core microbiome (Jami and Mizrahi, 2012; Petri et al., 2013b). Across studies there is general agreement that the core microbiome of cattle includes members of the phyla Firmicutes (especially genera *Ruminococcus* and *Butyrivibrio*) and Bacteroidetes (particularly genus *Prevotella*), along with some taxa present in lower abundance. Because each study used a relatively small number of animals, membership in the core microbiome would be expected to shrink upon inclusion of successively larger numbers of animals (i.e., upon generalization to a global population of hosts). Despite this, the core microbiome remains a useful concept because it focuses attention on the microbes that are likely to be either essential for, or at least major contributors to, overall ruminal fermentation. But we are again confronted with the familiar problems of assigning specific functions to the members of this community, whose cultured representatives often are nutritional generalists.

Once one gets beyond the core microbiome, it appears that there is extensive variability among individual animals with respect to the bacterial species composition of the rumen. Part of the variation is due to the presence of very low-abundance OTUs – often as singletons detected by NGS, but substantial inter-animal variation has been detected even when using relatively low-sensitivity, low-resolution methods such as automated riborsomal intergenic spacer analysis (ARISA, Welkie et al., 2010) or denaturing gradient gel electrophoresis (DGGE, Zhou et al., 2012). Host specificity in the rumen does not appear to be restricted to Domain Bacteria, as it has been observed for both the methanogenic archaeal and the protozoal communities (Zhou et al., 2012).

If the microbial community within each rumen is unique to its host, several questions arise: At what stage of life is this community assembled to the point where it can be regarded as compositionally and functionally unique? What environmental drivers determine the initial establishment and ultimate maintenance of each community? Can community composition and its resultant functionality be substantially altered by some combination of dietary manipulation and exogenous inoculation?

## Responses to Experimental Manipulation of the Ruminal Microflora

### Insights from Feeding Studies

A variety of microbial strains, some of them marketed commercially, have been tested as feed additives to improve animal performance, particularly in dairy and beef cattle. Much of this work has been summarized in excellent reviews (Yoon and Stern, 1995; Chaucheyras-Durand et al., 2008). Among the most heavily examined microbes have been *Saccharomyces* yeast, the filamentous fungus *Aspergillus oryzae* (AO), and lactic acid bacteria. In general, these strains have shown inconsistent production responses that are highly dependent on growth or lactation stage of the host, feeding level, and diet type. The few studies that have been conducted regarding persistence of the fed strains suggest that live cultures do not grow in the rumen, and in fact some of the products are as effective when added in non-viable forms (or in the case of AO, as a cell-free extract) as when added to the rations in live form (Yoon and Stern, 1995).

Even for strains that have shown occasional improvements in performance, it has proven difficult to convincingly demonstrate any of the proposed mechanisms of action. These proposed mechanisms range from consuming oxygen, to providing nutrients (e.g., vitamins or amino acids), to selectively enhancing growth of particular (usually unidentified) bacterial species. Based on our understanding of microbial ecological principles, the lack of persistence of the fed strains is not surprising, but the occasional demonstration of production improvement by direct fed microbials is sufficiently intriguing to warrant a more complete comparison of the microbial communities in animals fed these products versus control animals fed the same diet without the fed strains, using more modern techniques of molecular microbial ecology. There have been a few studies in this regard. For example, Mosoni et al. (2007) reported that sheep fed active dry yeast preparations along with a high-concentrate diet had enhanced levels of 16S rRNA corresponding to the cellulolytic bacteria *Ruminococcus albus* and *R. flavefaciens*. While the mechanism of action is not clear, it should be noted that these bacteria are among the most O_2_-sensitive ruminal bacteria in culture, and thus might benefit from the known O_2_-consuming capacity of the yeast. On the other hand, there is substantial evidence (discussed below) that cellulose digestion in the rumen is not limited by the number or activity of cellulolytic microbes, but by the accessibility of cellulose, so the effects on the cellulolytic community may be unrelated to the probiotic role of active dry yeast products, which may be complex and multifaceted.

### Insights from Ruminal Dosing Studies

A clear indication of the inertia and resilience of the ruminal community is provided by dosing studies with ruminally cannulated animals. In one of the earliest examples, Varel et al. (1995) isolated a strain of *Clostridium longisporum* from the rumen of a bison, and strain of *C. herbivorans* from the pig intestinal tract. Both bacterial strains were more actively cellulolytic in pure culture than were the common isolates of “classical” ruminal cellulolytic species (*Fibrobacter succinogenes, R. albus*, and *R. flavefaciens*), and on this basis it was hypothesized that these *Clostridium* strains would be able to be established in the rumens of animals fed diets high in cellulosic feeds. Varel et al. (1995) then dosed fermenter-grown cultures (6 l of culture, plus 20 l of buffer) into three ruminally cannulated cows whose rumens had been nearly completely emptied; after dosing the cows were returned to hay feeding. The inoculated strains had a distinctive colony pigmentation and morphology that allowed their easy identification on selective agar medium. Despite the massive inoculation into nearly emptied rumens, the dosed strains were cleared to undetectable levels, usually within 24 h of inoculation. While the inoculated strains were of ruminal or hindgut origin, they represented species that are not considered abundant in the rumen, which suggested that these strains were not highly competitive in the ruminal habitat from the start, and under the experimental conditions used, the quantitatively modest residual host community was easily able to displace the dosed species in relatively short order.

As summarized in **Table 5**, other reported dosing experiments with fibrolytic strains of ruminal origin have proven no more successful, even when using more modern and sensitive detection methods. It thus appears that the native (*autochthonous*) fibrolytic microbes within each individual rumen are sufficiently well adapted in their native habitat to outcompete non-native (*allochthonous*) strains or species introduced from other habitats, including other rumens. Within the ruminal habitat, competition is likely intensified by fiber limitation (i.e., although fiber concentration is high on concentration basis, most fiber is inaccessible).

**Table 5:** Ruminal dosing experiments with fibrolytic microbial strains.

Dosed strain	Source	Recipient animals	Result	Notes	Reference
*Clostridium longisporum* B6405 and *C. herbivorans* 54408	Bison (B6405), Pig (54408)	Three cannulated 6 years-old cows	Dosed strains not detected (<10^3^ Cells/mL) within 24--48 h of dosing	Rumen nearly emptied prior to dosing, and feeding resumed immediately after dosing.	Varel et al. (1995)
*Ruminococcus albus* (Y1, LP9155 or AR72), or *R. flavefaciens* (SY3 or AR67)	Lab strains	Total of 16 cannulated adult Merino sheep	Strains dosed daily for 9 days at 5 × 10^12^ cells/dose reached abundances of up to 6.5% of the bacterial community but did not persist.	No improvement observed in dry matter digestibility of Rhodes grass incubated *in situ* during dosing period.	Krause et al. (2001)
*R. flavefaciens* NJ + probiotic	Wild moose	Six cannulated non-lactating dairy cows	Dosed strain (6.8 × 10^11^ cells) did not persist.	Dosed strain declined by ~10^3^-fold within 24 h and was undetectable by 50 h after dosing.	Chiquette et al. (2007b)
		Calves (21--35 days old)	Dosed strain showed weak persistence.	Dosed strain detected at low levels (~10^2^ cells/mL) 7 days after cessation of dosing.	
*R. flavefaciens* 8/94-32	Norwegian reindeer	Three starved male reindeer	Dosed strain did not persist.	Population size of the abundant Ruminococcaceae family did not change. Some change in overall bacterial community composition observed.	Praesteng et al. (2013)
*R. flavefaciens* FD-1	Lab strain	Six lactating Murrah buffaloes	Equivocal results: Population of *R. flavefaciens* increased from 1.46 × 10^7^/mL prior to dosing to 2.52 × 10^7^/mL during the week after dosing concluded, but also increased in control buffaloes fed autoclaved cultures.	Very heavy oral supplementation of dosed strain [9 × 10^14^ cells (sic) on alternate days for 1 month].	Kumar and Sirohi (2013)
Ruminal fungi *Orpinomyces* sp. C-14 or *Piromyces* sp*.* WNG-12	Cattle	15 lactating Murrah buffaloes	Increased feed digestibility and up to 5.6% improvement in milk production.	Zoospore density higher in dosed animals, but level of dosage not reported.	Saxena et al. (2010)

The situation with non-fibrolytic microbes is not always as discouraging. Establishment of dosed strains has in a few cases been dramatically successful. Undoubtedly the premier example of a successful ruminal introduction is provided by the case of *Synergistes jonesii*. The tropical leguminous shrub *Leucaena leucocephala* contains high levels of the non-protein amino acid mimosine, which is ruminally converted to the goitrogenic 3,4-dihydroxypyridine. Jones and Megarrity (1986) noted that Indonesian goats were sensitive to mimosine poisoning, while Hawaiian goats were not. They further showed that a single oral dosing of ruminal contents from these resistant Hawaiian goats into Indonesian goats conferred resistance to mimosine poisoning. Allison et al. (1992) isolated the microbial agent, a novel bacterial species they named *Synergistes jonesii*, and demonstrated that the bacterium was only capable of using mimosine and two other amino acids as growth substrates. Subsequent studies revealed that inoculation of this bacterium either as a pure culture, or as ruminal contents from mimosine-resistant animals, readily conferred mimosine resistance to the recipient animals (Hammond, 1995).

A second example of a successful inoculation concerns toxicity of fluoroacetate, a secondary plant metabolite that blocks the action of citrate synthase, an essential enzyme of the tricarboxylic acid cycle. Gregg et al. (1998) successfully introduced a dehalogenase gene from a *Pseudomonas* strain into a ruminally derived strain of *Butyrivibrio fibrisolvens*, and then successfully established the recombinant strain by dosing into sheep, which subsequently exhibited retention of the strain over several months along with a markedly enhanced tolerance to fluoroacetate. In the case of both mimosine and fluoroacetate protection, inoculation success is clearly the result of the dosed strain filling an open niche, aided by the strain’s highly specialized metabolic capability and a substantial concentration of susceptible substrate. Interestingly, more recent work has revealed that fluoroacetate resistance appears to have developed naturally in some ruminal bacteria (Camboim et al., 2012).

Perhaps we should not be surprised by the general difficulty of establishing a single allochthonous microbial strain within a given rumen, unless that strain can fill an open niche (**Table 6**). At the risk of anthropomorphism, we may view the problem in more familiar sociological terms: In addition to abiotic stressors, an introduced strain is likely to encounter many enemies (direct competitors as well as their co-adapted symbionts, with whom they may have established productive mutualistic relationships worth defending) and few friends (unaffiliated microbes that might immediately benefit from cooperating with the introduced strain). This raises the question: can inoculation success be enhanced by introducing more complex assemblages, or even entire communities, of co-adapted species?

**Table 6:** Comparison of niche filling and niche replacement.

	Niche filling	Niche replacement
Initial status	Niche unoccupied	Niche occupied, sometimes by several competitors
Dosing and results	Single dosing often sufficient to establish dosed strain	Multiple doses sometimes (but often not) sufficient to establish dosed strain
Examples	*Synergistes jonesii* to impart resistance to mimosine toxicity	Most commercial probiotics; Experimental strains of fibrolytics and homoacetogens

### Insights from Exchanges of Ruminal Contents

The possibility that introduced strains can be established more effectively if accompanied by co-adapted community members can be examined via experiments in which ruminal contents are exchanged between pairs of ruminally cannulated animals. Early ruminal contents exchanges were conducted to test for specific physiological or nutritional outcomes rather than effects on the ruminal microbial community. Satter and Bringe (1969) exchanged ruminal contents between Holstein cows fed diets high in forages versus high in concentrates, and also switched the diets themselves coincident with the contents exchange. They observed that it took 5–6 days for the milk-fat depressing effects of the high-concentrate diet to be expressed in the recipient cow, and concluded that metabolic changes (including possible adaptation of the ruminal microbial community) were more important in controlling milk fat synthesis than were the amount and proportions of different VFA (acetate and propionate), which differed between the exchanged contents and would be expected to act more immediately. Interestingly, these researchers conducted additional comparative experiments in which abrupt dietary switches were performed without ruminal contents exchange, but they did not conduct ruminal contents exchanges while maintaining the cows on their separate diets. Cockrem et al. (1987) exchanged ruminal contents in varying amounts between cattle divergently selected for bloat susceptibility, and noted that susceptibility was determined primarily by ruminal contents volume (i.e., the extent to which the rumens were re-filled) rather than by the source of the added ruminal fluid. Cole (1991) demonstrated that exchange of ∼50% of ruminal contents through the cannulas of fed and fasted sheep (which would be expected to have high and low rumen microbial activity, respectively) had no measurable effect on feed intake or on various digestion parameters upon return of the animals to feed. These authors concluded that improving “rumen function” (including the ruminal fermentation) is unlikely to improve feed intake, and thus would not improve production. Taken together, these early studies suggested that the ruminal microbial community might be highly resilient and host-specific.

The above exchange studies demonstrated the adaptability of animals in response to dramatic changes in ruminal contents, but did not directly address the microbiology of the rumen prior to or following the exchange. More recent development of methods for characterizing the microbial community has permitted such examinations. Obviously it is not possible to sterilize the rumen prior to or during the exchange to remove the resident microbiota from the recipient animal, but it is possible to remove almost the entire contents of the rumen, leaving behind a modest portion (a few per cent) of the original resident community that can then be challenged with a relatively massive amount of a co-adapted microbial community from a donor animal. Such studies have shown that retention of even a small fraction of the original resident community is sufficient to facilitate (within days to weeks) a reassortment of the bacterial community to resemble that of the cow prior to contents exchange, even when the donor inocula are obtained from herd mates fed the same diet and housed under apparently identical conditions (Weimer et al., 2010a). One interesting and unexpected result of these exchange experiments was that ruminal pH and VFA profiles returned to those of the recipient cows much more quickly (within 1 day) than did the bacterial community composition. This indicates that the animal exerts ultimate control over its ruminal chemistry through salivary buffering and through absorption and passage of VFA, and in fact this may be one of the means by which the animal provides its own selective pressure on the microbial community. Future experiments could include the use of pairs of animals that are inherently more similar in ruminal chemistry, and could explore the response of all the major microbial groups (not just bacteria), with more detailed characterization using NGS.

## Microbial Ecology and the Prospects for Establishing Allochthonous Strains in the Rumen

The essential nutrient transformations within the rumen were identified in the 1950s and 1960s through the pioneering efforts of Hungate and others. From these studies it is apparent that, from an energy conservation and nutrient retention standpoint, the primary limitations to the ruminal fermentation are (1) the relatively slow rate and incomplete extent of fiber fermentation; (2) inefficient nitrogen utilization due to loss of feed protein by unproductive ruminal fermentation to ammonia; and (3) loss of feed energy to methane as a result of interspecies hydrogen transfer reactions. Ever since, rumen microbiologists have made these three limitations the foci of efforts to manipulate the ruminal fermentation.

Overall, there are two primary strategies for manipulating a given aspect of the ruminal fermentation: modifying the ration (including altering the proportions and compositions of the main ration ingredients as well as adding various chemical modifiers, enzymes, etc.), and directly altering the composition of the microbial community (primarily via feeding or addition of probiotics). This review shall consider only the latter strategy, with an emphasis on its likelihood for success based on our understanding of the principles of microbial ecology.

Dietary supplementation with probiotics has become fashionable in human nutrition, and is practiced to some extent in livestock agriculture in developed countries. The essential aim of probiotic feeding is to provide a more balanced and harmonious gut microbial community that improves a suite of gut functions including digestion and immune response. In many cases, probiotic strains appear to function transiently in the community and their effects are sustained only by frequent (daily or continuous) feeding – a marketing executive’s dream comes true. Successfully establishing a probiotic strain would require that the strain surmount numerous challenges: surviving the stresses of delivery and inoculation (culture storage, air exposure, mixture into feeds); locating and accessing its food source within the rumen; competing effectively against community members already well-established to the unique characteristics of an individual ruminal habitat; avoiding predation and antagonistic agents (e.g., bacteriocins); establishing productive mutualistic interaction with particular community members; and growing at a rate sufficient to exceed the dilution rate of ruminal contents.

The general characteristics of the ruminal habitat and the associated challenges in establishing an allochthonous microbe therein parallel in many ways those of another familiar and economically important habitat, the anaerobic sludge digester used in biological waste treatment. In both habitats, a complex mixture of substrates of high organic matter content are degraded under semi-continuous conditions by a dense and diverse microbial community that operates at several trophic levels. Because of the large number of potential interactions among community members, establishing a new member in the community would likely require a number of accommodations by the existing community, and a failure of any one of these may be sufficient to prevent retention and establishment of the new member. Thus it is instructive that there are few reports (Duran et al., 2006; Lu et al., 2009) describing the successful introduction of a probiotic microbial strain into the sludge digester community, and these have only been demonstrated at laboratory scale under which reactor conditions were controlled over a relatively narrow range.

### Enhancing Fiber Fermentation

Enhancing plant cell wall digestibility in the rumen by microbial intervention has proven remarkably difficult. Plant fiber is a complex matrix of cellulose, hemicellulose, and lignin, along with smaller amounts of pectins and protein. Lignin itself is essentially indigestible under anaerobic conditions, but its effects do not end there. It also serves as a physical barrier that limits the accessibility of ruminal microbes and their fibrolytic enzymes to otherwise digestible structural polysaccharides. Digestibility can be enhanced by pretreatments (e.g., ammonia) that act primarily by removing lignin or disrupting its chemical bonding to hemicellulose, but such pretreatments generally are not cost effective for ruminant agriculture. Ruminants rely instead on an effective physical pretreatment (mastication and rumination) to improve the digestibility of relatively indigestible plant fiber, largely by increasing available surface area for enzymatic and microbial attack (Van Soest, 1994). There is ample experimental evidence that structural polysaccharide degradation follows first-order kinetics with respect to substrate concentration (Waldo et al., 1972; Van Soest, 1973), and its rate is limited by available surface area (Weimer et al., 1990), and not by the abundance and activity of the microbial population. In fact, several studies have revealed that the *in vitro* digestion rate of both neutral detergent fiber and cellulose does not decline until ruminal contents are diluted approximately five- or sixfold (Pell and Schofield, 1993; Mouriño et al., 2001).

If the rate and extent of fiber digestion are limited by substrate accessibility, one would expect that artificial augmentation of the ruminal community by exogenous addition of fibrolytic microbes would not discernibly improve fiber digestion, and dosing experiments by and large have supported this expectation. Most studies have been aimed specifically at quantifying persistence of dosed strains, without any reported measure of animal performance. Of these, the only report (Chiquette et al., 2007b; **Table 4**) in which dosing of a fibrolytic bacterial strain was associated with maintaining population sizes of >10^7^ cells/mL for the represented species beyond the dosing period, used an aggressive (and frankly incredible) dosing schedule (reportedly 9 × 10^14^ cells per dose on alternate days over a 30 days period). In cases where fiber digestibility has been measured, dosing has not improved digestibility or animal performance even during periods of frequent dosing (Krause et al., 2001). These results are in accord with the work of Dehority and Tirabasso (1998), who showed that increasing the cell density of cellulolytic bacteria in the rumen 10-fold by feeding a diet high in purified wood cellulose did not improve *in situ* digestibility of alfalfa fiber.

Recent work with ruminal fungi suggest that this microbial group may show promise as a probiotic for improved fiber digestion (Puniya et al., 2014). Ruminal fungi typically account for only a few per cent of total microbial biomass in the rumen, but their unique ability to combine physical disruption of plant tissue by the growing appresoria, with enzymatic hydrolysis of cell wall polysaccharides may increase in available surface area of fiber that currently limits its rate of digestion in the rumen. Several early studies showed very modest improvement in nutrient digestibility upon dosing with ruminal fungi, but strain persistence was not measured. However, Saxena et al. (2010) reported modest improvement in milk production, as well as increased ruminal zoospore densities, in water buffalo dosed weekly for 180 days with two different fungal strains, (**Table 4**); unfortunately, the specific abundance of the individual species was not measured.

### Decreasing Nitrogen Losses

Under modern agricultural production conditions, feed nitrogen (primarily in the form of protein) is inefficiently used by ruminants. It is primarily excreted as ammonia, which acts variously as an air pollutant and a substrate for nitrification to nitrate, which is both a water pollutant and a substrate for respiratory denitrification that yields nitrous oxide, a potent greenhouse gas, as an ancillary product. Use of biological approaches to decrease ruminal protein degradation have received relatively little attention, in part because non-biological interventions are available (e.g., roasting of soybeans to reduce protein reactivity), and in part because there appear to be few viable biological approaches. Decreasing ruminal proteolysis is challenging because the functionally redundant ruminal community contains many species that produce a variety of proteases, and that further ferment amino acids and di- and tripeptides. Some species can potentially be more troublesome than others. Pure cultures of some obligate amino acid fermenters (sometimes called hyper ammonia-producing bacteria, HAB) have specific rates of ammonia production up to 100-fold higher than do *Prevotella* species (Russell, 2002). However, the overall contribution of the HAB may be minor, as the classical HAB species (*Clostridium sticklandii, Clostridium aminophilum,* and *Peptostreptococcus anaerobius*) seem to comprise only a small fraction of the ruminal bacterial community [<0.7% of 16S rRNA gene copy number, as determined by qPCR (Wallace, 1996), while *Prevotella* is perhaps the most abundant genus in the rumen (Stevenson and Weimer, 2007)].

### Methane Mitigation

Methane is the most fully reduced form of carbon, and thus its production is an ideal electron sink for balancing fermentations of organic matter in the rumen and other microbial habitats. However, enteric methanogenesis in ruminants represents a loss of 2–14% of feed energy, and is widely regarded as a substantial contributor to anthropogenic greenhouse gas emissions (McAllister et al., 1996). Much effort has been expended in investigating strategies for redirecting the flux of reducing equivalents in the rumen toward alternative electron acceptors, either through the use of chemical agents that inhibit methanogens or their symbionts, or through the direct addition or feeding of various exogenous electron acceptors. The chemical inhibitors often work well *in vitro* but show smaller and more transient effects *in vivo* (McAllister et al., 1996). Alternative electron acceptors (e.g., nitrate, sulfate, or fumarate) can potentially be more effective on a long-term basis, but their use *in vivo* is not practical due their high cost or toxicity issues (particularly H_2_S produced by sulfate reduction), and the potential requirement for additional inoculation of a direct-fed microbial (Jeyanathan et al., 2014). An alternative approach is to directly inhibit ruminal methanogens and allow the microbial community to redirect electron flow to other acceptors, especially C_3_–C_6_ VFA. However, careful *in vitro* product balance studies with methane inhibited cultures do not yield a full accounting of this electron flow (see Ungerfeld, 2015, for a detailed meta-analysis), suggesting that it may be necessary to employ additional interventions to realize significant methane mitigation.

One such strategy that has long interested microbiologists is to promote reductive acetogenesis (4H_2_ + 2 CO_2_ → CH_3_COOH + 2 H_2_O) in the rumen, which would enhance the retention of feed energy in a form readily utilizable by the host. However, ruminal methanogens successfully fill the niche of H_2_ utilization in the rumen, and their kinetic and thermodynamic advantages over known acetogens are well established from both pure culture comparisons, and mixed culture studies conducted *in vitro* (Fievez et al., 1999). On this basis, one could predict that acetogens would not establish themselves when exogenously added into the rumen. *In vitro* experiments have largely confirmed these expectations: Acetogens are known to colonize the rumen early in development (Morvan et al., 1994) and can be sustained in gnotobiotic lambs, but quickly disappear upon colonization by methanogens (Gagen et al., 2012). Perhaps the most interesting aspect of the methanogenesis/acetogenesis story is the fact that, as pointed out by Jeyanathan et al. (2014) acetogens can outcompete methanogens in the gut environments of some non-ruminant animals ranging from termites to wallabies to humans. Understanding the basis of competitive success of acetogens in these species may inform future efforts to mitigate methanogenesis in ruminants (Wright and Klieve, 2011).

Because of its intimate link to decreased feed efficiency, there have been some efforts to characterizing the methanogenic community of animals that differ in feed efficiency. Studies have revealed that beef steers grouped as low- or high-efficiency (high and low residual feed intake, respectively) differed in the relative proportions of individual methanogenic OTUs (viz., higher OTU diversity and higher densities of *Methanospaera stadtmanae* and *Methanobrevibacter* sp. strain Amb4 in less efficient animals), even though the total density of methanogenic cells did not differ (Zhou et al., 2009). How these proportional differences may be related to differing methane emissions among animals is not clear. Do particular methanogenic OTUs differ in methane output per unit of cell mass? Can efficiency be increased by ridding the community of specific OTUs?

Answering these questions will ultimately require isolation of individual methanogenic OTUs and performing quantitative comparisons of growth and product formation. In the meantime, culture-independent methods can provide useful perspective on how methane emissions might be de-coupled from the abundance of the total methanogenic population. Poulsen et al. (2013) have recently shown that dietary supplementation with rapeseed oil (RSO, 33 g/kg DM) decreased methane emissions from dairy cows by 6.2% and substantially decreased the relative population size of one specific group of methanogens, the relatively abundant Methanomassiliicoccales (formerly Rumen Cluster C – Thermoplasmata; Iino et al., 2013). Metatranscriptomic data suggest that this group (no representatives of which have been isolated) are methylotrophs that utilize either methylamines (MA) or methanol as methanogenic substrates, and that transcription of two Methanomassiliicoccales-specific methyl-CoM reductase genes decreased upon RSO supplementation. Ruminal concentrations of MA were significantly higher under RSO supplementation (0.22 mM vs. 0.04 mM without supplementation), suggesting that MA (possibly produced from choline and related compounds) are normal methanogenic precursors *in vivo*. Because the flux and passage of MA in the rumen is not known, it is not yet clear if these cellular-level decreases are sufficient to completely account for the observed decrease in methane emitted by the animal, nor is it clear whether the Methanomassiliicoccales may have additional capabilities to produce methane from other feed components. Regardless, this study reinforces the notion that the methanogens are not a physiologically monolithic group, and that methane mitigation strategies might be productively directed toward specific methanogenic subpopulations. Alternatively, Shi et al. (2014) have reported that, while sheep that produce high or low amounts of methane have similar levels of methanogens, expression of certain methanogen-specific genes is elevated in the higher-emitting animals. This suggests that strategies for mitigating methane emissions might be expanded to include regulation of gene expression rather than attempts to control methanogen population sizes *per se*.

### Other Potential Microbial Interventions

Ruminal acidosis resulting from sudden increases in the amount or form of dietary starch is a serious health issue that also reduces productivity in both dairy and beef cattle. Acidosis can occur in both an acute and sub-acute form, and is typically ascribed to the activity of bacterial species such as *Streptococcus bovis* that produce lactic acid as a major fermentation end product (Russell, 2002). Lactic acid has a pK_a_ of ∼3.8 and is thus a much stronger acid than are the volatile fatty acids (pK_a_∼4.8). Use of probiotic bacterial strains that consume lactic acid has been proposed as a means of attenuating acidosis, with particular efforts focused on *Megasphaera elsdenii* strain NCIMB 41125 (extensively reviewed by Meissner et al., 2010). Dosing experiments conducted on dairy cows at various stages of lactation and levels of production and fed different diets have yielded inconsistent effects on ruminal pH and on milk production and composition, although decreases in ruminal lactate concentrations were observed. Because relatively few ruminal microbial species are known to actively ferment lactate, it would seem that lactate utilization might provide *M. elsdenii* with an opportunity to fill an available niche in the rumen, but additional studies are necessary to quantify persistence of the dosed species. Interestingly, qPCR studies have revealed that *M. elsdenii* is barely detectable in most ruminal samples tested, although its abundance was strongly elevated under conditions of both subacute acidosis (Khafipour et al., 2009) and milk fat depression (MFD; see Palmonari et al., 2010; Weimer et al., 2010b; Mohammed et al., 2014). Further research is needed to determine if this species has a causative role in MFD, or if it is merely associated with MFD through some other primary driver.

A second strategy to control acidosis is via probiotic addition of bacteria that rapidly ferment starch but do not produce appreciable amounts of lactic acid. Chiquette et al. (2007a) ruminally dosed *Prevotella bryantii* 25A daily for a 10 week period (3 week prepartum to 7 week postpartum) into cows fed a diet whose forage:concentrate ratio was 40:60. Relative to control cows, lactate concentrations in the dosed cows were halved (0.7 mM vs. 1.4 mM), VFA concentrations were higher, and there was a tendency toward increased milk fat percentage. Interestingly, ruminal ammonia was also elevated in the dosed strains, suggesting perhaps that the dosed strain contributed to undesirable degradation of protein and its hydrolytic products. Unfortunately the abundance or persistence of the dosed strain in the rumen was not quantified.

### Practical Considerations for Probiotics

For any manipulation involving a probiotic, identification of the candidate microbial agent through laboratory and animal trials is just the beginning. Additional research is necessary to develop a formulation that retains activity over the course of shipping, storage and application under production conditions. A large body of information on formulation (often proprietary) has been gathered for specific microbial products in other applications (probiotics for human use, silage inoculants, etc.), and formulation technology has been developed for certain ruminal probiotics (particularly yeast and lactic acid bacteria). However, probiotic products based on microbes isolated from the rumen itself may face additional challenges due to their often strictly anaerobic nature, poor viability retention in stationary phase, and lack of a highly resistant resting state (spores, cysts, etc.) to resist environmental insult. Perhaps the most detailed formulation research on a true ruminal microbe has been obtained with *Megasphaera elsdenii*, noted above for its potential to control lactic acidosis (Meissner et al., 2010). This bacterium has been successfully packaged into a pouch containing separate compartments for dried cells and growth medium; squeezing of the pouch combines the ingredients at the point of use in a readily incubated form under anaerobic conditons. Once grown, the culture was dosed into the rumens of cannulated cows in research studies, but it is not clear how effectively it can be introduced into animals via feeding.

## Concluding Remarks

From the above discussion, several salient points emerge. First, the microbial community is phylogenetically diverse, metabolically redundant, and both compositionally and functionally resilient. Second, the stability and host specificity of the community provide substantial barriers to manipulation of community composition and function. Third, successes in modifying the microbial community to improve animal performance have thus far been most dramatic for specialist microbes that fill an otherwise unoccupied niche. Fourth, recent advances in determining community composition and diversity have far outpaced our ability (or willingness) to dissect out the physiological and ecological roles of individual phylotypes, particularly those that impact animal performance. Elucidating these roles and exploiting them to manipulate the composition and function of the ruminal microbiome represents probably the most challenging and most important means by which microbiologists can advance the productivity, profitability, and sustainability of ruminant animal agriculture.

## Conflict of Interest Statement

The author declares that the research was conducted in the absence of any commercial or financial relationships that could be construed as a potential conflict of interest.
